# Is it feasible and ethical to randomize patients between surgery and non-surgical treatments for gastrointestinal cancers?

**DOI:** 10.3389/fonc.2023.1119436

**Published:** 2023-03-16

**Authors:** Artur Rebelo, Johannes Klose, Jörg Kleeff, Ulrich Ronellenfitsch

**Affiliations:** Department of Visceral, Vascular and Endocrine Surgery, University Hospital Halle (Saale), Martin-Luther-University Halle-Wittenberg, Halle, Germany

**Keywords:** cancer, randomized control trial (RCT), ethical, gastrointestinal, oncology

## Abstract

**Background:**

In several settings in the treatment of gastrointestinal cancers, it is unclear if the addition of surgery to a multimodal treatment strategy, or in some circumstances its omission, lead to a better outcome for patients. In such situations of clinical equipoise, high-quality evidence from randomised-controlled trials is needed to decide which treatment approach is preferable.

**Objective:**

In this article, we outline the importance of randomised trials comparing surgery with non-surgical therapies for specific scenarios in the treatment of gastrointestinal cancers. We explain the difficulties and solutions of designing these trials and recruiting patients in this context.

**Methods:**

We performed a selective review based on a not systematic literature search in core databases, supplemented by browsing health information journals and citation searching. Only articles in English were selected. Based on this search, we discuss the results and methodological characteristics of several trials which randomised patients with gastrointestinal cancers between surgery and non-surgical treatments, highlighting their differences, advantages, and limitations.

**Results and conclusions:**

Innovative and effective cancer treatment requires randomised trials, also comparing surgery and non-surgical treatments for defined scenarios in the treatment of gastrointestinal malignancies. Nevertheless, potential obstacles to designing and carrying out these trials must be recognised ahead of time to avoid problems before or during the trial.

## Introduction

In several settings in the treatment of gastrointestinal cancers, the available data cannot answer the question whether surgery or non-surgical treatments lead to a better outcome for patients. In such situations of clinical equipoise, to provide a valid answer to this question, as for any treatment recommendation in medicine, high-quality evidence is needed. Despite improvements in the quality of clinical research in surgical oncology, several aspects regarding the design of studies comparing surgery to no surgery are still a problem. Only some surgical treatments have been assessed in randomised controlled trials (RCTs), and a relevant proportion of surgical treatments is based on scarce and conflicting evidence (1). Practical and personal experience drives the apparent progress in surgery to a much higher extent than in drug treatments. Surgical RCTs represent only 15% of the published RCTs, and only about 24% of surgical therapies are supported by evidence from RCTs (2–4). A large proportion of published studies in surgical oncology have a retrospective observational design with several limitations and inherent risk of bias. Despite of recent efforts in designing surgical RCTs, in a systematic review of 388 randomised clinical trials, the sizes of surgical trials were small (5). Also, discrepancies with the published protocol and reporting bias were frequent (6–11). Randomising patients between additional surgery and no surgery involves confronting several problems: commercial interests in the light of high reimbursements for many surgeries, lack of cooperation between surgical and non-surgical departments, hesitancy and ethical concerns of patients and investigators to randomise between surgery and non-surgical treatments with the knowledge that surgery is a viable option, and blinding of patients and surgeons.

In this article, we outline the importance of conceiving randomised trials comparing surgery with non-surgical therapies for specific scenarios in the treatment of gastrointestinal cancers, highlighting the difficulties and solutions of designing these trials and recruiting patients in this context.

## Why do we need randomization between surgery and non-surgical treatments?

An RCT has several advantages. The prospective nature of the study implies a planned assessment, documentation, and follow-up (1). The blinded RCT provides the highest level of evidence in evidence-based medicine and minimizes bias. Randomization is the best design to establish causal relationship between exposure and outcome. Non-randomized comparative cohort studies provide important data, but only with statistical adjustments (a. e. propensity score analysis) from covariates, an association between intervention and outcome may be shown, and a considerable risk of bias persists.

Regarding RCTs comparing surgery and non-surgical treatments, different types of comparison groups are possible: no active intervention, medical management, deferred surgery, active monitoring (“watch and wait”), physical or manual therapy and placebo (sham surgery).

In a systematic review comparing quality domains in trials of surgical interventions to a previously reported control sample of trials of medical interventions, although reporting of quality domains was suboptimal, surgical trials compared favorably to medical trials (12). “*They were 24% more likely to have an adequate method of random sequence generation, and 71% more likely to have an adequate method of allocation concealment. However, blinding was 40% less likely to be adequate in surgical trials, and sources of funding were 33% less likely to be reported*” (12). Although it is not a specific limitation of RCTs, publication bias is also a problem that has to be faced when designing these trials. Selective outcome reporting is a known problem of RCTs (10). For example, in neurosurgery, it was shown that RCTs comparing surgical to non-operative treatment fairly frequently changed their outcome measures, which may distort the available results of a given trial und undermines the trials’ credibility (13).

Randomized controlled trials comparing surgery with non-surgical treatments are rare, but with the development of new multimodal therapy regimens in gastrointestinal cancer surgery, randomized comparisons of different medical and surgical approaches are needed (14). For example, conversion surgery is defined as an operation aiming to clear all tumor sites after tumors that had initially been considered technically unresectable or where a resection was deemed to be of no oncological benefit, responded to chemotherapy and become resectable (15). Another example is the possible omission of surgery after very good response to chemotherapy or chemoradiotherapy, as it is now discussed regarding complete response after total neoadjuvant therapy for rectal cancer (16).

## Advantages and disadvantages of non-randomized trials

Usually, observational studies have some advantages when compared to RCTs: lower cost, greater timeliness, and a broader range of patients eligible for study inclusion. Despite its limitations on comparing treatments, they are used to identify risk factors and prognostic factors (17). Furthermore, in some clinical scenarios, non-randomized prospective cohort studies categorizing and comparing observational data may represent better alternatives than RCTs (18–21). These types of studies potentially lead to a higher participation of the patients in the interventional group, mostly according to the preferences of the clinician or the patient. Despite of the risk of selection bias, these studies give insights on the outcomes of the effects of surgical treatments and provide, in some cases, quality evidence comparable to RCTs. The level of evidence gained from a poor quality RCT is not necessarily better than that from a well-conducted cohort study. *A priori* registration of protocols is still not required in observational studies but would be a major strength to avoid explorative data analyses. Conducting and reporting observational studies according to the Strengthening the Reporting of Observational studies in Epidemiology (STROBE) Statement is a requirement for publication in some journals (22). Nevertheless, prospective observational studies usually represent complementary evidence or are the basis for designing RCTs (23, 24). Chalmers et al. reported that 56 percent of non-randomized trials reported on favorable treatment effects, as compared with 30 percent of blinded, randomized-controlled trials. This potential selection bias was also reported in other studies (25–28).

As demonstrated, the potential for bias in RCTs is normally lower when compared to non-randomized studies. Bias is defined as the systematic difference between the study results and an RCT, addressing the same question and conducted on the same participant group that had no flaws in its conduct. Assessing bias of a non-randomized study involves comparing it to a hypothetical pragmatic RCT that compares the health effects of the same interventions and is conducted in the same participants without features putting it at risk of bias. The assessment of risk of bias in non-randomized studies involves pre-intervention, at-intervention, and post-intervention features of the study (29, 30). The bias related to non-random allocation results in over- or underestimations of treatment effects, being large enough to lead studies to false conclusions. Even when applying case-mix adjustment methods (i. e. logistic regression, propensity score) bias stays significant (31). The absence of reliable methods to prevent the biasing consequences of selection bias in observational research leaves non-randomized studies for situations when RCTs are unfeasible or unethical. Unfeasibility of RCTs usually is present when the disease or indication is very rare, and ethical problems often arise when very large treatment effects can already be seen in non-randomized studies, so that equipoise can no longer be assumed (32).

## Disadvantages of randomized trials

Surgical trials are difficult to conceive, and only half of the initiated trials reach their recruitment target (33–35). When performing these studies, surgical clinician scientists face several obstacles such as the surgical learning curve and the lack of financial support. Furthermore, blinding problems, poor generalizability of the trial population and difficulties with randomization in emergency situations represent important adversities that researchers must overcome. These and other problems result in 21% of RCTs in surgery being discontinued and 34% being unpublished (36, 37)

Surgical trials face patient and surgeon related challenges: a radical choice between treatments, patients’ discomfort with randomization between an operation and no operation, patients’ or clinicians’ *a priori* preferences for one or the other treatment, and an imbalanced presentation of the treatment options to patients (38). Regarding trials comparing surgical and non-surgical interventions, slow recruitment is mentioned to be the most common problem that researchers have to confront, with the consequence that no evidence-based treatment recommendations can be made (39–42).

Furthermore, historical and cultural limitations are relevant when designing RCTs comparing surgery with non-surgical treatments. Most surgical treatments were developed to treat conditions that were untreatable with other means and were potentially life-threatening. Once a surgical treatment is established, it is difficult and sometimes appears ethically questionable to compare it to a medical treatment or surveillance. Structural, political, and commercial aspects also play an important role. Regarding ethical aspects, the possible adverse effects of surgery and non-surgical treatments usually differ substantially, and surgery is mostly irreversible with organs or parts thereof being removed. Due to these limitations, an indirect selection bias may be present in these RCTs, as only a small subgroup of patients may agree to participate on them.

Placebo controlled trials represent another option in this context. In the context of surgery, placebo means sham surgery, i.e. general anesthesia without an actual operation, or a surgical procedure intended to mimic the actual operation. However, the conception of a placebo control in a surgical RCT may be challenging and ethically difficult because the surgical unlike the medical “placebo” bears a relevant degree of invasiveness. If there is no expected benefit (beside the placebo effect), patients are usually resistant to undergo the low-risk anesthesia required for a sham surgery intervention. Blinding is also very difficult in this kind of trials. Nevertheless, surgical RCTs with a placebo arm are feasible, with the recruitment of patients remaining the leading challenge (43).

## Advantages of randomized trials

Notwithstanding the challenges outlined above, randomized-controlled trials remain the gold standard for generating evidence on what is the best treatment for a given condition or in a specific setting. This holds equally true with regard to both medical treatments as well as surgical procedures and is of particular importance for patients with gastrointestinal cancers, where the choice of treatment has direct implications on survival, treatment-related morbidity and mortality, and quality of life, among other outcomes. Therefore, all reasonable efforts should be made to design and carry out randomized-controlled trials also for comparing surgical treatments with no surgery in patients with gastrointestinal cancers. Motivating patients for enrolling into such trials requires open, patient-centered, and evidence-based communication. Only by thoroughly explaining all expected risks and benefits, both in terms of procedural and long-term oncological outcomes, in an impartial way, patients can be empowered to make an informed decision on trial participation, which will ultimately enhance the probability of enrollment (44, 45). In a situation of assumed clinical equipoise, which is the foundation of all RCTs, surgery should neither be regarded only as a chance for cure or prolongation of life without appreciating its associated risks nor as a mere invasive procedure with morbidity and mortality risks without considering possible beneficial effects on oncological outcomes like survival. Quality of life, which can possibly be affected in both a positive and negative direction by a surgical procedure, is of high importance for many patients when deciding for or against surgery and must be specifically addressed in such conversations (46). Pre-existing preferences of patients towards one or the other treatment need to be considered, addressed openly and discussed using available evidence (47). The general advantages of participating in a controlled clinical trial, such as close monitoring, possibly more frequent follow-up visits and access to novel treatments, need to be well explained to patients, but should not be overstated in a promotional manner (48). While discontinuation of the trial by single patients should obviously not be encouraged, the freedom of choice to quit trial participation at any time, and eventually even to seek the alternative treatment, i.e. surgery for patients who had been randomized into the no surgery arm or no surgery for patients who had been randomized into the surgery arm (as long as the operation has not been carried out) should be addressed, too. “Placebo”-controlled trials are almost impossible to realize in surgical oncology. Sham surgery, which would potentially delay further non-surgical treatments such as chemotherapy, seems ethically not acceptable for cancer patients. Sham anesthesia could be a theoretical less invasive option, but a lack of scars would still render long-term blinding of patients not feasible. Therefore, RCTs in surgical oncology including those enrolling patients with gastrointestinal cancer are usually open-label studies.

Given that the likelihood of selective participation in RCTs randomizing between surgery and no surgery based on patients’ characteristics is considerable, efforts should be made to collect baseline but also outcome data from patients who are screened and offered trial participation, but who ultimately choose not to enroll. Observational cohorts comprising patients who refused trial participation or did not meet all inclusion criteria but were treated with identical interventions as if they had participated in the respective trial, can support evidence generated by RCTs. In a specific example of an RCT comparing preoperative radiotherapy plus surgery with surgery alone in patients with retroperitoneal sarcoma, results from such an observational cohort closely resembled the results from the actual RCT (49).

Another possible solution is the use of adaptive randomized trial designs. This allows modifications to the trial design during the collection of patient outcome data and despite its challenges, may present several advantages when compared to standard trial designs (50).

## Examples of successful randomization between surgery and non-surgical treatments

Several examples show that RCTs comparing surgery and no surgery in specific treatment settings of gastrointestinal cancers can be successfully conducted.

In a potentially curative setting, the FFCD 9102 trial randomized patients with thoracic esophageal squamous cell carcinoma or adenocarcinoma who had shown clinical response to neoadjuvant chemoradiotherapy to either resection or continuation of chemoradiotherapy (51). Only 14 of 273 patients (5.1%) fulfilling all eligibility criteria refused randomization. Compliance with the allocated treatment was high with only 10 of 129 patients (7.8%) randomized to surgery deciding against the operation and only 1 of 130 patients (0.8%) randomized to continuation of chemoradiotherapy demanding surgery. A trial with a similar design randomized 37 of 38 eligible patients (97.4%) with squamous cell carcinoma of the esophagus, who showed complete clinical and metabolic response to chemoradiotherapy, to esophagectomy or observation (52). While all 18 patients allocated to observation were compliant with that treatment with some patients being operated on later because of secondary progression, 6 of 19 patients (31.6%) allocated to surgery chose not to have the operation. Overall enrolment into the trial was much slower than expected which together with the low compliance with treatment in the surgery arm led to premature trial closure. The trialists assumed that compliance of patients allocated to surgery was low due to the timing of randomization after complete response had been confirmed and with a general change of local treatment patterns towards observation instead of surgery. The ongoing RENAISSANCE trial randomizes patients with oligometastatic gastroesophageal adenocarcinoma and no disease progression following chemotherapy between additional chemotherapy or resection of the primary tumor and the metastatic lesions followed by chemotherapy (53). In a similar population, i.e. patients with gastric adenocarcinoma and one metastatic site, the REGATTA trial randomized between gastrectomy followed by chemotherapy and chemotherapy alone (54). All enrolled 175 patients were successfully randomized. While 7 of 86 patients (8.1%) allocated to chemotherapy alone withdrew consent, 1 of 89 (1.1%) patients allocated to gastrectomy plus chemotherapy decided not to undergo the operation.

In rectal adenocarcinoma, which often shows very good or even complete response to neoadjuvant chemoradiotherapy, several trials randomizing between rectal resection and organ preservation, either through a watch-and-wait strategy or local excision, have been or are being conducted. The GRECCAR-2 trial randomized 145 out of 146 eligible patients (99.3%) who demonstrated good response to chemoradiotherapy (55). Only 1 of 74 patients (1.4%) allocated to local excision underwent rectal resection while 8 of 73 patients (11.0%) allocated to rectal resection underwent local excision and 3 of 73 patients (4.1%) no surgery at all. In the TREC trial, 55 of 152 identified eligible patients (36.2%) consented to randomization between organ preservation by transanal microsurgery and radical rectal resection (56). Of the 27 patients allocated to organ preservation, 3 patients (11.1%) crossed over to the rectal resection arm, and one patient had to end protocol treatment because of metastatic disease. Of the 28 patients allocated to rectal resection, 3 patients (10.7%) refused surgery and crossed over to the organ preservation arm.

The SYNCHRONOUS trial randomized patients with colon cancer and unresectable synchronous metastases to resection of the primary before starting chemotherapy (187 patients) and chemotherapy without prior resection (206 patients). Results have so far only been published in abstract form, and no information on the proportion of eligible screened patients who were randomized and on compliance with the allocated treatments are available (57).

A Chinese trial randomized patients with metastatic gastrointestinal stromal tumor responding to imatinib treatment either to surgery of residual disease followed by continuation of imatinib treatment or to continuation of imatinib treatment without surgery (58). Although only 5 of 46 screened eligible patients (10.9%) refused entering the trial, the trial had to be closed prematurely due to slow accrual. However, all patients received the treatment they were allocated to with no crossing over or refusal of therapy.

## Conclusions

As in all other fields of medicine, guidelines, and recommendations for when and if surgery for gastrointestinal cancers should be performed need to be based on evidence of the highest possible level. Such evidence can only be provided by well-designed RCTs with other study designs bearing a non-negligible risk of bias, which compromises the validity of their results. A randomization between an operation and no operation with either a watch-and-wait approach or an alternative non-surgical treatment is ethically fully acceptable if there is clinical equipoise between the two treatments. However, it is often more difficult for patients and physicians to accept than a randomization between two drugs or even between a presumably active drug and a placebo. Frequently, there is an *a priori* preference towards either the surgical treatment or against surgery, even if such preferences are not supported by available data. A dedicated explanation of all expected risks and benefits associated with trial participation, and the open discussion of patients’ pre-existing preferences are key factors for achieving fast and unselected recruitment into these RCTs. Several trials conducted in esophageal cancer and colorectal cancer show that randomization between surgery and no surgery or microsurgery can be successfully done both in a setting with curative intent and in metastatic disease. These examples should be encouraging for researchers to conceive of, design, and carry out more of these RCTs to provide high-level evidence for unanswered treatment questions for gastrointestinal cancers.

## Author contributions

AR, JKlo, JKle and UR performed research, selected manuscripts wrote and edited the paper. All authors contributed to the article and approved the submitted version.
